# Whole‐exome sequencing identified novel compound heterozygous variants in a Chinese neonate with liver failure and review of literature

**DOI:** 10.1002/mgg3.1515

**Published:** 2020-11-18

**Authors:** Zailong Qin, Qi Yang, Shang Yi, Limei Huang, Yiping Shen, Jingsi Luo

**Affiliations:** ^1^ Genetic and Metabolic Central Laboratory Guangxi Birth Defects Research and Prevention Institute Maternal and Child Health Hospital of Guangxi Zhuang Autonomous Region Nanning Guangxi China; ^2^ Department of Genetics Harvard Medical School Boston MA USA

**Keywords:** hyperlactatemia, hypoglycemia, liver failure, TRMU, whole‐exome sequencing

## Abstract

**Background:**

Liver failure caused by TRMU is a rare hereditary disorder and clinically manifests into metabolic acidosis, hyperlactatemia, and hypoglycemia. Limited spectrum of TRMU pathogenic variants has been reported.

**Methods:**

Whole‐exome sequencing was employed for the diagnosis of a 5‐day‐old female who suffered from severe neonatal hyperlactatemia and hypoglycemia since birth. Sanger sequencing was performed to confirm the origin of the variants subsequently. Variants classification was followed to ACMG guideline.

**Results:**

A compound heterozygosity of a frameshiftc.34_35dupTC (p.Gly13fs) and a missense c.244T>G (p.Phe82Val) in TRMU was detected, both variants are novel and pathogenic. Analysis of clinical and genetic information including patients reported previously indicated that there is no significant correlation between the genotype and the phenotype of TRMU‐caused liver failure.

**Conclusion:**

To the best of our knowledge, this is the first case report of TRMU‐caused liver failure in China. Whole‐exome sequencing is effective for conclusive diagnosis of this disorder and beneficial for its clinical management.

## Graphical abstract

Liver failure caused by TRMU is a rare hereditary disorder. Here, we report the first case of TRMU‐related liver failure in China. We suggest that TRMU deficiency should be considered when ALF was presented with hyperlactatemia, hypoglycemia, and metabolic acidosis in infants. l‐cysteine and N‐acetylcysteine should be considered in clinical treatment of patients diagnosed with TRMU mutation.

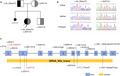

## 1 INTRODUCTION

Acute liver failure (ALF, MIM613070) is generally defined as loss of liver function resulting from rapid injury or death of majority of hepatocytes and occurring within 8 weeks of onset of symptoms (Lee, 1993). Neonatal acute liver failure (NALF) is included in a narrow range of ALF which the insult may undergo before the development of the liver and threaten fetal life (Taylor & Whitington, 2016). The etiology of NALF is multifaceted, including metabolic disorders, neonatal hemochromatosis, viral infections, etc (Squires, 2008; Squires et al., 2018). Nevertheless, some NALF patients are diagnosed as indeterminate cause. To be noticed that metabolic defects, especially mitochondrial hepatopathies (MH) is increasingly considered to be a prominent cause of NALF which presents deficiencies in respiratory complexes (including I, III, and/or IV) or mitochondrial DNA depletion (Lee & Sokol, 2007). Up to now, several pathogenic variants of MH related to NALF have been reported recently, including DGUOK, POLG, MPV17, TWINKLE,TRMU, SUCLG1, BCS1L, SCO1, etc (Squires et al., 2018).

TRMU (OMIM610230) is located in 22q13 and consists of 11 exons. This gene encodes tRNA 5‐methylaminomethyl‐2‐thiouridylate methyltransferase, an essential enzyme specifically for the 2‐thiolation of the wobble position of the mitochondrial tRNA‐Glu, tRNA‐Gln, and tRNA‐Lys (Sasarman et al., 2011). The main domains of TRMU protein include adenine nucleotide alpha hydrolase‐like domains, rossmann‐like alpha/beta/alpha sandwich fold, and tRNA 5‐methylaminomethyl‐2‐thiouridylate methyltransferase domain which is involved in the biosynthesis of the modified nucleoside 5‐methylaminomethyl‐2‐thiouridine (mnm5s2U) present in the wobble position of some tRNAs (Hagervall et al., 1987). The posttranscriptional modification by TRMU enables accurate synthesis and optimal function of the 13 respiratory chain subunits with the presence of cysteine. Hepatic TRMU pathogenic variants were first described in 13 cases of infantile liver failure (Zeharia et al., 2009). So far, different types of TRMU pathogenic variants have been discovered in several cases of NALF among different ages (Enns et al., 2000; Gaignard et al., 2013; Schara et al., 2011; Uusimaa et al., 2011). Those patients with severe symptoms caused by TRMU pathogenic variants had chance to undergo spontaneous recovery, which could be possibly explained by increased level of l‐cysteine (Boczonadi et al., 2013, 2015). However, there is not enough published data to reveal the mechanisms of TRMU pathogenic variants in development and spontaneous improvement of NALF. Here, we present the case of a 5‐day‐old girl with hypoglycemia as the first symptom since birth and genetically diagnosed as ALF with novel compound heterozygous TRMU pathogenic variants by whole‐exome sequencing. To the best of our knowledge, this is the first case report of ALF caused by TRMU pathogenic variants in China.

## 2 MATERIALS AND METHODS

### 2.1 Sample collection and DNA extraction

The proband was died at 5 days after birth in local hospital. There left no blood or tissue sample except dried blood spot for neonatal screening. In this circumstance, proband's genomic DNA was isolated from dried blood spot using Lab‐Aid DNA kit (Zeesan Biotech Co., Ltd, Xiamen, China).

### 2.2 Whole‐exome sequencing and data analysis

Genomic DNA was extracted from dried blood spot and captured to create the library for whole‐exome sequencing by Agilent SureSelect Human Exon V5 kit (Agilent Technologies, Santa Clara, CA) according to the manufacturer's protocol. The libraries were sequenced by HiSeq X Ten with PE150 strategy (Illumina, San Diego, CA) with a read depth over 120X and more than 95% of the targeted regions covered over 20X. The sequencing reads were mapped to the Genome Reference Consortium Human genome build 37 (GRCh37). The Genome Analysis Toolkit (GATK) was used for variant calling. TGex software (LifeMap Sciences, Alameda, CA) was used to annotate the variants. Transcript NM_018006 was used as the reference sequence. The TRMU variants and their origins were verified by Sanger sequencing. All the variants were classified according to ACMG/AMP guidelines.

### 2.3 Proband and clinical characteristics

The proband was a female born at 39+ weeks gestation by vaginal delivery weighing 2660 g and 49 cm body height. No detected abnormality was found during gestation. Her Apgar score was 10‐10‐10. She presented with poor feeding, weak cry, low response, irregular respiration, and mild tetany at 10 hours after birth. After another 3 hours, she presented with no cry and no response. Glucose monitoring indicated sever neonatal hypoglycemia (1.64 mmol/L, normal 3.9–6.1). Apart from hypoglycemia, laboratory test also indicated metabolic acidosis (pH 6.80, normal 7.35–7.45. HCO‐ 4.10 mmol/L, and normal 21–28), hyperlactatemia (15.00 mmol/L and normal 0.5–1.7), and disseminated intravascular coagulation (PT‐T45.50 sec, normal 9.4–12.5; FIB 0.57 g/L, normal 2.0–4.0; INR 4.24, normal 0.87–1.16; APTT 126.80 sec, normal–28‐41;TT31.70 sec, and normal 10.3–16.6). Protein synthesis and liver enzymes indicated that liver damage has occurred (TP 52.50 g/L, normal 60–80; ALT 30.00 U/L, AST 139.00 U/L, normal 0–40). According to the clinical manifestations and laboratory test results, mechanical ventilation was employed at once for the proband. Glucose injection was performed, and dexamethasone therapy was used to maintain the blood glucose levels. Dopamine therapy was used to improve metabolic acidosis. Ceftazidime was used to anti‐infection. Heparin was injected to prevent thrombosis. Fresh frozen plasma was injected for coagulation factor supplementation. However, the treatment was not effective, especially for metabolic acidosis. She developed with encephalopathy and fell into deep coma. Finally, she died due to respiratory failure after 5 days. The family history was negative.

### 2.4 Variant analysis

Whole‐exome sequencing identified novel compound heterozygous pathogenic and likely pathogenic variants in the alleles of TRMU: c.34_35dupTC (p.Gly13fs)/c.244T>G (p.Phe82Val) which inherited from her father/mother. According to the ACMG/AMP guidelines, c.34_35dupTC was classified to be pathogenic (one very strong evidence PVS1, one moderate evidence PM2, and one supporting evidence PP4) and c.244T>G was classified to be likely pathogenic (two moderate evidence PM2, PM3 and three supporting evidencePM5_supporting, PP3, PP4). The PM5_supporting was used as a supporting evidence because of the record of c.246C>G (Accession: VCV000393172.2) in ClinVar database.

## 3 DISCUSSION

The 5‐methylaminomethyl‐2‐thiouridylate methyltransferase encoded by TRMU is a conserved functional protein during the modification of mitochondrial tRNA specific for glutamate, lysine, and glutamine (Yan et al., 2005). The modification is required for efficient function of mt‐tRNA in protein synthesis. TRMU is highly expressed in tissues with high metabolic rates including heart, liver, kidney, and brain (Yan et al., 2006). It has been hypothesized that TRMU protein might be involved in the assembly of enzyme complexes containing iron‐sulfur clusters that involved in complex II of the respiratory chain (Sasarman et al., 2011). Loss‐of‐function variants in this gene may cause 5‐methylaminomethyl‐2‐thiouridylate methyltransferase activity and tRNA binding insufficient, leading to mitochondrial translation defect and decrease protein synthesis that result in liver failure, hypoglycemia, hyperlactatemia, etc.

TRMU‐related liver failure was first described by Zeharia et al in 2009 (Zeharia et al., 2009). The patients presented ALF in infancy are subject to routine laboratory investigations and revealed elevated liver transaminases, hyperlactatemia, hypoglycemia, coagulopathy, etc. Pathogenic variants in TRMU were identified in these patients, but the underlying mechanisms remain unclear. Functional research in a hepatocyte‐specific Mtu1 (human TRMU homolog) knockout mice model revealed that Mtu1^−/−^ mice were embryonic lethal while Mtu1^+/−^ mice can survive. However, Mtu1^+/−^ mice exhibited mt‐tRNAs thiolation deficiency, mitochondrial translation impairment, sever disruption of mitochondrial membrane integrity, and decrease in respiration complex activities in the hepatocytes (Wu et al., 2016). Another research in mtu1 knockout zebrafish model also demonstrated that mtu1 deficiency cause the global decreases of mitochondrial tRNAs. The aberrant mitochondrial tRNA metabolisms resulted in the impairment of mitochondrial translation, respiratory phenotypes, and reductions of mitochondrial ATP production (Zhang et al., 2018).

In our case, the proband was a female infant. She was lethargic at 4 hours after birth. Laboratory investigations showed that she had severe hypoglycemia and hyperlactatemia. Abnormal liver enzymes, coagulation disorders, and hepatic encephalopathy indicated that liver failure has occurred. However, symptomatic treatment was ineffective, and she was died after 5 days. Whole‐exome sequencing identified novel compound heterozygous pathogenic and likely pathogenic variants of TRMU with proband.

Based on the currently reported clinical information of patients with TRMU‐caused liver failure, the features are highly variable and no explicit correlations between clinical manifestations and genotype have been clarified yet. Therefore, to elucidate the underlying relationship between the genotype and the phenotype of TRMU‐related liver failure, we analyzed the genetic and clinical information of all the reported patients and our case, and the summary can be found in Table 1. Routine laboratory investigations revealed that hyperlactatemia was commonly presented in all patients (100%, 24/24) followed by hepatomegaly (70.83%, 17/24), while splenomegaly was less presented (12.5%, 3/24). In addition, elevated alpha‐fetoprotein (83.33%, 20/24), hypoglycemia (45.83%, 11/24), cholestasis (20.83%, 5/24), jaundice (75%, 18/24), coagulopathy (75%, 18/24), vomiting (66.67%, 16/24), and poor feeding (70.83%, 17/24) were also presented in patients. Of note, elevated lactate was the most prominent symptom. According to our summary, three of patients were reportedly underwent spontaneously recover (12.5%, 3/24), but seven patients died including our case (29.17%, 7/24).

**TABLE 1:** Genetic and clinical features of the liver failure patients caused by TRMU pathogenic variants

Patient	Age	Variants	Liver failure	Hepatomegaly	Splenomegaly	Hyperlactatemia	Elevated alpha‐fetoprotein	Cholestasis	Hypoglycemia	Jaundice	Coagulopathy	Hypotonia	Vomiting	Poor feeding	Outcome	ALT	AST	GGT	Lactate	Reference
1	4 months	c.232T>C (p.Tyr77His)	Y	—	—	Y	Y	—	Y	—	—	—	—	—	NA	NA	NA	NA	NA	Gil‐Margolis et al. (2018)
2	4 months	c.232T>C (p.Tyr77His)	Y	—	—	Y	Y	—	Y	—	—	—	—	—	NA	NA	NA	NA	NA	Gil‐Margolis et al. (2018)
3	4.5 months	c.835G>A (p.Tyr77His), c.1037_1040delTCAA (p.Leu346 fs)	Y		—	Y	—	—	Y	—	Y	—	—	—	NA	NA	NA	NA	NA	Grover et al. (2015)
4	3 months	c.835G>A (p.Tyr77His), c.248+1G>A	Y	Y	Y	Y	Y	Y	Y	Y	—	Y	Y	Y	D	4ULN	8ULN	6ULN	5↑	Gaignard et al. (2013)
5	4 months	c.835G>A (p.Tyr77His), c.649G>A (p.Glu217lys)	Y	Y	Y	Y	Y	Y	Y	Y	Y	—	—	—	RS	13ULN	17ULN	11ULN	10.0↑	Gaignard et al. (2013)
6	2 days	c.697C>T (p.Leu233Phe)	Y	Y	Y	Y	Y	Y	Y	Y	—	Y	—	—	RS	2ULN	5ULN	N	6.0↑	Gaignard et al. (2013)
7	2 years	c.711_712insG (p.Gln238 fs), c.1081_1082insAGGCTGTGC (p.Arg361insAlaValArg)	Y	Y	—	Y	Y	—	Y	Y	—	Y	Y	—	R	1346↑	1310↑	1000↑	7.0↑	Schara et al. (2011)
8	6 months	c.232T>C (p.Tyr77His)	Y	Y	NA	Y	Y	—	NA	Y	Y	NA	Y	Y	NA	367↑	NA	356↑	5.5↑	Zeharia et al. (2009)
9	4 months	c.232T>C (p.Tyr77His)	Y	Y	NA	Y	Y	—	NA	Y	Y	NA	Y	Y	NA	169↑	NA	621↑	4.5↑	Zeharia et al. (2009)
10	2 months	c.232T>C (p.Tyr77His)	Y	Y	NA	Y	Y	—	NA	Y	Y	NA	Y	Y	NA	1150↑	NA	—	20↑	Zeharia et al. (2009)
11	3 months	c.232T>C (p.Tyr77His)	Y	Y	NA	Y	Y	—	NA	Y	Y	NA	Y	Y	NA	293↑	NA	139↑	6.6↑	Zeharia et al. (2009)
12	4 months	c.232T>C (p.Tyr77His)	Y	Y	NA	Y	Y	—	NA	Y	Y	NA	Y	Y	NA	417↑	NA	—	7.0↑	Zeharia et al. (2009)
13	4 months	c.232T>C (p.Tyr77His)	Y	Y	NA	Y	Y	—	NA	Y	Y	NA	Y	Y	NA	430↑	NA	—	20↑	Zeharia et al. (2009)
14	3 months	c.232T>C, c.706‐1G>A	Y	Y	NA	Y	Y	—	NA	Y	Y	NA	Y	Y	D	400↑	NA	157↑	30↑	Zeharia et al. (2009)
15	6 months	c.697C>T (p.Leu233Phe), c.28G>T (p.Ale10Ser)	Y	Y	NA	Y	Y	—	NA	Y	Y	NA	Y	Y	NA	532↑	NA	305↑	3.2↑	Zeharia et al. (2009)
16	1 months	c.835G>A (p.Tyr77His), c.500_510del (p.Ala167 fs)	Y	Y	NA	Y	Y	—	NA	Y	Y	NA	Y	Y	D	1193↑	NA	77↑	19↑	Zeharia et al. (2009)
17	6 months	c.815G>A (p.Gly272Asp)	Y	Y	NA	Y	Y	Y	NA	Y	Y	NA	Y	Y	NA	—	NA	—	—	Zeharia et al. (2009)
18	1 days	c.40G>A (p.Gly14Ser)	Y	Y	NA	Y	Y	—	NA	Y	Y	NA	Y	Y	NA	1146↑	NA	270↑	20↑	Zeharia et al. (2009)
19	1 days	c.2T>A (p.Met1Lys)	Y	Y	NA	Y	Y	—	NA	Y	Y	NA	Y	Y	D	93↑	NA	—	7.0↑	Zeharia et al. (2009)
20	2 days	c.2T>A (p.Met1Lys)	Y	Y	NA	Y	Y	—	NA	Y	Y	NA	Y	Y	D	229↑	NA	—	10.0↑	Zeharia et al. (2009)
21	3 months	c.304A>G (p.Asn102Asp), c.835G>A (p.Tyr77His)	Y	—	—	Y	Y	Y	Y	Y	Y	—	—	Y	NA	450↑	NA	420↑	75.0↑	Indolfi et al. (2017)
22	4 days	c.117G>A (p.Trp39*), c.680G>C (p.Arg227 Thr)	Y	—	—	Y	—	—	Y	—	Y	—	—	Y	D	309↑	1228↑	861↑	19.9↑	Soler‐Alfonso et al. (2019)
23	5 weeks	c.117G>A (p.Trp39*), c.680G>C (p.Arg227 Thr)	Y	—	—	Y	—	—	Y	—	—	—	Y	—	NA	347↑	540↑	624↑	41.9↑	Soler‐Alfonso et al. (2019)
24	5 days	c.34_35dupTC (p.Gly13 fs), c.244T>G (p.Phe82Val)	Y	—	—	Y	—	—	Y	—	Y	Y	—	Y	D	42↑	139↑	NA	15.0↑	Our case

Abbreviations: ↑, because normal range was different in literatures, the ↑ symbol was used to indicate the detection value higher than the normal range; D, death; N, normal; NA, not available; R, recovery; RS, recovered spontaneously; ULN, upper limit of normal; Y, yes.

**FIGURE 1:**
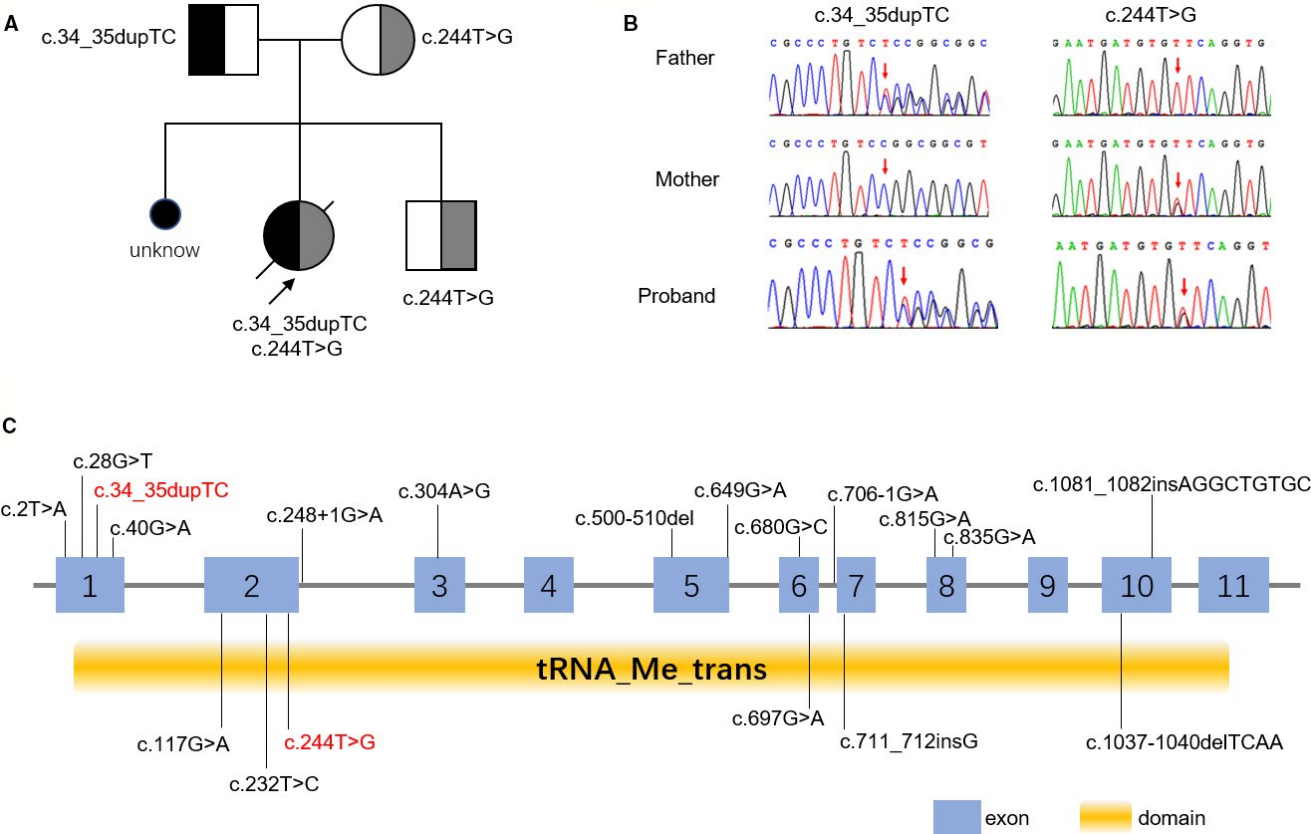
Pedigree analysis and variants distribution of TRMU. (a) Pedigree analysis of patient's family. Pathogenic variant c.34_35dupTC was paternally inherited and c.244T>C was maternally inherited. (b) Variants identification by sanger sequencing. (c) The variants previously reported are shown in the schematic according to their chromosomal location. Our case was shown in red font. TRMU conserved domain was predicted by NCBI Conserved Domain Database and Pfam database

Total of 19 variants in TRMU have been confirmed in these patients. Missense was the most detected variant (57.89%, 11/19). In addition, missense c.232T>C (p.Tyr77His) was detected most frequently (Table 1), and those patients with such missense are mostly Yemenite Jewish, indicating a founder effect. However, there was no significant correlation among variant type, variant position, protein domain, and patient outcome (Figure 1, Table 1). It may be due to the limited sample size and more cases need to be collected for further investigation.

It has been reported that treatment with supplementation of l‐cysteine and N‐acetylcysteine might be helpful to liver functional recovery of infants diagnosed with TRMU deficiency (Boczonadi et al., 2015; Soler‐Alfonso et al., 2019; Zeharia et al., 2009). Soler‐Alfonso C *et al* have reported two siblings with hepatopathy due to compound heterozygous of TRMU (c.117G>A, c.680G>C). The elder one was died of related complications. However, when the younger one was found to have the same type of TRMU pathogenic variant by prenatal diagnosis, he was treated with l‐cysteine and N‐acetylcysteine supplementation once he was born. This treatment strategy was effective to prevent the increase of liver enzymes and lactic acidosis as l‐cysteine act as sulfur donor which is essential to maintain TRMU normal function and the respiratory chain complex activities (Boczonadi et al., 2013, 2015; Soler‐Alfonso et al., 2019).

In conclusion, TRMU‐related liver failure is a life‐threatening disease that causes neonatal death, although only a few may recover spontaneously. Appropriate precautions for TRMU‐caused liver failure are urgently needed in recessive hereditary disease prevention. Carrier screening in pre‐pregnancy or prenatal diagnosis is believed to be very effective in recessive disorder prevention (Rose & Wick, 2018). Nowadays, with fast development of high‐throughput sequencing, carrier screening and prenatal diagnosis can be achieved rapidly, for example, whole‐exome sequencing adopted in our case.

We suggest that TRMU deficiency should be considered when ALF was presented with hyperlactatemia, hypoglycemia, and metabolic acidosis in infants (Schara et al., 2011). l‐cysteine and N‐acetylcysteine should be considered in clinical treatment of patients diagnosed with TRMU pathogenic variants (Boczonadi et al., 2015; Soler‐Alfonso et al., 2019; Zeharia et al., 2009).

## CONFLICT OF INTEREST

The authors declare that they have no conflict of interest.

## AUTHOR’S CONTRIBUTIONS

Collection of clinical data: Zailong Qin and Jingsi Luo. Data analyzed and interpreted: Zailong Qin, Qi Yang, Shang Yi, Limei Huang, and Yiping Shen. Writing and review of original draft of the manuscript: Zailong Qin and Jingsi Luo.
